# Editorial: Papillary thyroid cancer: prognostic factors and risk assessment

**DOI:** 10.3389/fendo.2025.1578271

**Published:** 2025-03-03

**Authors:** Vincenzo Marotta, Luca Scafuri, Jacopo Manso

**Affiliations:** ^1^ Unità Operativa Complessa (UOC) Clinica Endocrinologica e Diabetologica, Azienda Ospedaliera Universitaria (AOU) San Giovanni di Dio e Ruggi d’Aragona, Salerno, Italy; ^2^ Oncology Unit, “Andrea Tortora” Hospital, Azienda Sanitaria Locale (ASL) Salerno, Pagani, Italy; ^3^ Associazione Oncology Research Assistance (O.R.A.) Ente Terzo Settore (ETS)-Oncology Research Assistance, Salerno, Italy; ^4^ Endocrinology Unit, Azienda sanitaria Universitaria Friuli Centrale (ASUFC), Oncology Area Department, University-Hospital S. Maria della Misericordia, Udine, Italy

**Keywords:** papillary thyroid cancer, thyroid neoplasms, thyroid nodules, prognosis, risk stratification

## Introduction

Thyroid cancer is not only the most common endocrine malignancy, but its incidence has been continuously growing during the last 40 years, being more than triplicated (1). Among thyroid malignancies, papillary thyroid cancer (PTC) is by far the most common, reaching a prevalence of about 80%, and, notably, represents the unique responsible for the increased incidence (2). Upon thyroid ablation (thyroidectomy with or without iodine-131 administration), PTC has excellent prognosis with nearly 100% 5-years disease-specific survival (3) and very low risk of disease recurrence (4). However, 25-30% of patients experience persistent structural disease/recurrence upon initial standard treatment, and a relevant portion of them (11 and 57% for those showing lymph node (LN) and distant metastases, respectively) die as related to PTC (5).

In such scenario, to identify the PTC subgroup with more aggressive behaviour and poorer outcome represents the prognostic goal.

Historically, the risk stratification was based on a limited number of static parameters (6), available at the time of initial treatment, whom prognostic significance had been mainly weighted by retrospective analyses, intrinsically carrying a relevant bias likelihood. Based on the combination of such features, death-predicting [such as the AJCC/TNM (7)] and persistence/recurrence predicting [such as that provided by the American Thyroid Association (ATA) (5)] systems were built, with the latter being the most applied in clinical practice. However, when tested into real-life, these approaches revealed suboptimal long-term risk stratification, due to the low [less than 30% (4)] proportion of variance explained (PVE) [a statistical measure analysing the capability of a staging system to predict the outcome of interest (8)], and, more importantly, to the low positive predictive value (PPV) (9), which impairs the identification of the high-risk patients.

In order to overcome these limitations, the majority of guidelines (10, 11) elaborated a prognostic dynamic model, where disease evolution, as assessed by post-ablative biochemical and morphological data, was added to static parameters. According to such approach, long-term PTC management is determined by the so-called response to initial therapy assessment, a dynamic evaluation based on the determination of disease status starting 6-18 months after thyroid ablation, and updated at each follow-up visit. The incorporation of such parameter in the prognostic staging has demonstrated dramatic improvement of the risk stratification power (4).

However, an improvement of PTC risk stratification, as based on initial clinico-pathological features, is still required for optimizing clinical management, especially in some challenging settings, such as the heterogeneous category of subjects at intermediate risk of recurrence and the micro-PTC.

Aim of the present Research Topic was to refine the risk assessment of PTC, also focalizing on specific pathological features and PTC settings.

## Overviews of the contributions

Overall, 20 articles were published within the Research Topic. One of the most relevant was the study by He et al., which was aimed at improving the initial prognostic stratification, as performed at the immediate post-operative time and therefore based on static clinico-pathological characteristics. Authors applied the decision tree methodology on a large amount of differentiated thyroid cancer patients (mainly composed of PTC) from the SEER database, in order to define a more accurate staging for the prediction of cancer-specific survival (CSS), as compared with the latest AJCC/TNM update [8^th^ Edition (7)]. By means of such approach, a new TNM system was proposed, characterized by better metrics (higher PVE and area under the curve), as compared with the AJCC/TNM.

A number of contributions were focused on the prediction of PTC-related LN metastases (Yoon et al., Zhang et al., Sun et al., Chen et al., He et al.). This is a crucial item, as 30% of PTC patients experience LN involvement, and this event may worsen prognosis, especially in case of lateral macro-metastatic disease (12). Among the mentioned body of papers, we consider of great relevance the study by Yoon et al., which focused on a cohort of micro-PTC, typically characterized by indolent behaviour and even advisable to active surveillance (13). Authors identified a series of pre-operative ultrasonography (US) parameters (extra-thyroidal extension (ETE), multiplicity, upper lobe tumour location, and non-parallel shape) as independent predictors of lateral LN metastasis, therefore providing clinicians useful insights for choosing between surgery and surveillance. Remarkable findings were also provided by Zhang et al., who searched for risk factors of LN spread in PTC patients aged ≥ 65 years. Since age historically represents a predictor of poor survival in PTC, elderly patients have to be considered by definition a high-risk subgroup (14). The most relevant finding of the paper was the independent relationship of a series of clinico-pathological features (male gender, tumour size ≥ 1cm, age ≥ 70, and microcalcifications) with lateral LN metastases, which may allow clinicians to select “very high risk” patients in the context of a high risk category.

Two contributions (Lu et al., Xu et al.) were focused on the most challenging PTC prognostic category, namely the ATA intermediate risk of disease recurrence. This involves heterogeneous cases, from PTC with minimal ETE to those with LN metastases, and prognostics as well as clinical management, particularly attaining the indication to perform radioiodine (RAI) ablation, are not clearly codified (15). Lu et al. found that more than 5 central LN metastases and higher pre-RAI stimulated thyroglobulin (Tg) levels were independent predictors of non-excellent response (not cured disease) after complete thyroid ablation (surgery + RAI). Xu et al. focused on PTC at intermediate risk, as defined by the presence of LN metastases. They found that tumour size, multifocality, concomitant autoimmune thyroiditis, metastatic LN rate, and pre-RAI stimulated Tg were independent predictors of response to complete thyroid ablation.

Ultimately, two contributions (Wang et al., Kim et al.) dealt with pediatric differentiated thyroid carcinoma, where PTC prevalence is even higher, as compared with adults. This represents a hot-topic in thyroid oncology, due to the increasing incidence and the more advanced stage at diagnosis, as compared with adult PTC. We consider as of great relevance results from the Wang et al.’s study, where authors identified a set of independent predictors of disease cure upon complete thyroid ablation: T stage, pre-RAI stimulated Tg, and response to initial treatment [as defined by the ATA guidelines (16)].

## Implications and future directions

This Research Topic provides news insights about the prediction of specific pathological features affecting PTC outcome, such as the development of LN metastases, and about the risk assessment in the context of specific clinical settings, such as elderly and pediatric PTC, micro-PTC, and PTC at intermediate risk of recurrence.

The main limit of the studies included in the Research Topic, similarly to the vast majority of publications about PTC prognostics (which represent the mainstay of the current guidelines), is the retrospective nature. Hence, there is a great need of data from prospective observational studies, in order to refine the actual impact of each clinical features on disease outcome and to improve the risk assessment tools.
